# Improving Printed and Thermoformed Conductors on Polycarbonate with a Thin-Film BNNT Interlayer for Next-Generation In-Mold Electronics

**DOI:** 10.1021/acsami.5c07261

**Published:** 2025-09-09

**Authors:** Kaitlin Wagner, Arnold J. Kell, Xiangyang Liu, Liliana Gaburici, Joseph Manion, Chantal Paquet, Benoît H. Lessard

**Affiliations:** † Chemical and Biological Engineering, 120472 University of Ottawa, 161 Louis Pasteur, Ottawa, Ontario K1N 6N5, Canada; ‡ Quantum and Nanotechnologies Research Centre, 423375 National Research Centre Canada, 100 Sussex Drive, Ottawa, Ontario K1A 0R6, Canada; § School of Electrical Engineering and Computer Science, University of Ottawa, 800 King Edward Avenue, Ottawa, Ontario K1N 6N5, Canada

**Keywords:** thermoforming, boron nitride nanotubes, polycarbonate, molecular ink, current-carrying capacity, thermal management

## Abstract

The processes of thermoforming 2D-printed electronics into 3D structures can introduce defects that impact the electrical performance of conductors, making them more susceptible to thermal failure during high electrical power/current applications on temperature-sensitive substrates. We therefore report the use of a thin-film boron nitride nanotube (BNNT) interlayer to directly reduce heat stress on linear and serpentine metallic traces on polycarbonate substrates thermoformed to 3D spherocylindrical geometries at varying elongation percentages. We demonstrate that the BNNT interlayer helps to improve the electrical conductivity of highly elongated thermoformed 3D traces in comparison to traces on bare polycarbonate. Further, we correlate localized substrate thinning at high elongation areas with increases in the local trace resistance. These resistance increases create localized “hot spots” in the traces when high voltages and currents are applied to them. BNNT interlayers provide thermal protection to the underlying substrate and enable them to endure localized temperatures 1.5 times higher than those on bare substrates, as high currents are applied to the silver traces. Overall, this study demonstrates the use of BNNT interlayers as valuable thermal management materials to facilitate the development of more reliable and higher-performing conductive metal traces for use in 3D electronics and in-mold electronics applications.

## Graphical abstract

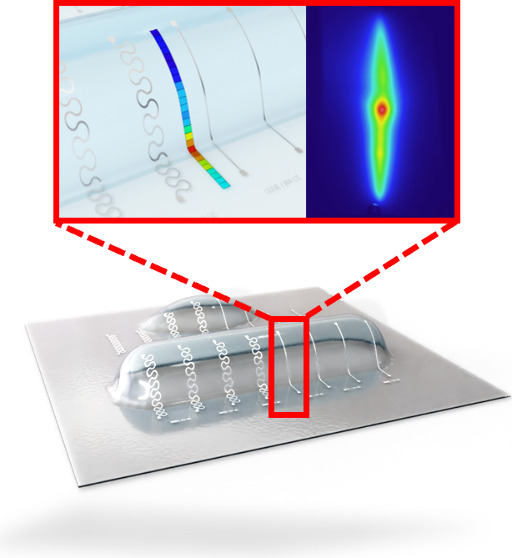

## Introduction

Recent interest in 3D electronics has garnered motivation to move away from complex and costly printing techniques that deposit inks directly on 3D parts, such as aerosol jet and multiaxial direct ink writing, to low-cost in-mold electronics fabrication.^1,2^ When optimized using techniques with low-cost and low-energy requirements, the resulting 3D architectures enable new unconventional applications that can expand how printed electronics are designed and integrated into everyday items for the automotive,^3–5^ aerospace,^6^ packaging,^7–9^ and medical device industries.^10,11^

In-mold electronics (IME) enable the manufacturing of 3D electrical features without having to physically print in the third dimension. In one approach, IME uses thermoforming to convert heat-molded 2D substrates that have printed traces on their surfaces into 3D shapes. Inks are typically screen-printed onto a substrate, dried, and subsequently brought above their glass transition temperature (*T*_g_) in a thermoforming machine before being vacuum-formed over a positive mold until they cool.^12^ Traces printed onto the substrate are stretched alongside the substrate because they accommodate the change in shape and volume transitioning from 2D to 3D. Traces printed with traditional flake- and particle-based inks rely on contact of the metal particles to ensure a conductive feature; however, as the substrate stretches during the thermoforming process, cracks can form in the trace, breaking the metal–metal contact and rendering the trace nonconductive post-thermoforming.^13^ This tends to occur more frequently when elongation of the traces exceeds 40–50%. Molecular inks (MINKs) are a class of conductive inks that are formulated with metal precursors. These inks are printed such that the metal precursor remains in its molecular state but can be processed into a metallic film during the thermoforming process. The advantage of using MINKs during thermoforming is that the ink trace will remain partially liquid as it undergoes the 2D-to-3D transition, allowing the ink to flow, remain crack-free, and maintain its conductivity following conversion to a 3D silver trace.^13^ It must be noted that there are specific locations where the substrate and traces elongate during the thermoforming process. Both the substrate and traces in these elongated areas are thinner than the surrounding areas, resulting in localized areas of higher resistance. This can cause localized temperature increases when large currents are applied to 3D circuits, and this bottleneck results in the trace material overheating and eventually melting/burning the underlying substrate, rendering the trace nonconductive.^14^ Interlayers have been previously incorporated into printed device structures to improve performance,^15–17^ thermal capacities,^18,19^ and mechanical integrity;^20,21^ however, their benefits have not been extensively studied in the context of 3D electronics produced from thermoforming techniques. In recent studies, nanothin interlayers of boron nitride nanotubes (BNNTs) were shown to improve the current-carrying capacity (CCC), overall thermal management capabilities, and mechanical durability of 2D-printed electronics structures.^19,22^ In the context of thermoforming, these BNNT properties should provide a level of thermal protection for the substrate, where they can reduce heat accumulation in areas where the conductive trace has thinned, creating hot spots during use and therefore improving the trace operability in high-current applications. Thermoforming also causes strain on the traces because of the elongation required to produce 3D features from the original 2D structures. We have previously demonstrated that incorporating BNNT interlayers between plastic substrates and conductive traces produced from MINKs enables the traces to better accommodate mechanical stresses related to repeated bending/flexing in comparison to the bare substrate. It is also of interest to understand whether similar mechanical benefits are present for thermoformed MINK traces on BNNT interlayers and lead to improvements in the electrical properties of 3D traces.

This work reports the first use of a BNNT interlayer as a mechanical and thermal barrier to improve the electrical properties of thermoformed 3D silver MINK traces printed on polycarbonate (PC) substrates. The high thermal conductivity and mechanical durability of the random network of BNNTs provide reinforcement to the printed traces upon deformation and elongation during the thermoforming process. The thermal dissipative properties of the BNNT network reduced hot-spot formation at elongation points for the thermoformed traces, leading to higher CCCs and improved functionality for 3D IME devices.

## Experimental Methods

### BNNT Dispersion and Deposition

Boron nitride nanotube (BNNT) material^200^ and powdered poly­(vinyl butyral) (PVB) at a 1:1 wt % ratio were added to ethanol as a green-solvent stable BNNT dispersion, leading to a final BNNT concentration of 2.0 mg/mL and a total dispersion volume of 1.0 L. Details about the dispersion fabrication are available in work completed by Wagner et al.^23^ This PVB/BNNT solution was spray-coated onto clean, 12 in. × 12 in. polycarbonate (PC) substrates using an Iwata Eclipse HP-CS Gravity Feed Dual Action Airbrush and a IS850 Smart Jet Compressor. During the spray-coating process, the substrate was placed on a heated platform set to 50 °C to assist in solvent evaporation. To ensure an even coating of nanotubes, the entire substrate was coated by using a back-and-forth motion while moving down the substrate to avoid overlap. Once the solvent was dried, the substrate was rotated 90° and the process was repeated until 6 layers of nanotubes were deposited onto the substrate. Additional trials with 4-, 8-, and 10-layer samples were investigated; however, 6 layers proved to be the desired thickness. The 6-layer film maximized thermal distribution while visually exhibiting a more uniform film post-thermoforming, leading to optimized integrity and morphology of both the BNNT film and trace upon thermoforming. In comparison, the 4-layer film had poor heat distribution, as previously demonstrated,^19^ and the 8-layer BNNT film led to significant disruptions perpendicular to the direction in which the substrate was stretched, particularly at the harsh elongation points.

### Screen Printing and Thermoforming

Silver (Ag) molecular ink (MINK) was prepared by using silver oxalate and an amino-based carrier. Further details about the ink synthesis and characterization can be found in work previously published by Liu et al.^13^ The ink was stored at −15 °C and warmed to room temperature prior to printing. When the ink reached room temperature, it was screen-printed onto both bare and BNNT-coated PC substrates. After printing, the samples were immediately passed three consecutive times through a UV conveyor (America Ultraviolet, 170 cm conveyor length) fitted with a gallium lamp and an iron lamp, as described by Liu and co-workers.^13^ Both lamps were set to midlevel intensity, and the conveyor speed was set to 30 feet/minute (FPM). Irradiance data for one pass under both lamps at 30 FPM were measured using an EIT UV Power Puck II radiometer and are tabulated in Table S1. Samples were thermoformed directly after UV processing using a Formech 450DT thermoformer. Traces printed on PC substrates, both bare and BNNT-coated, were placed under the thermoformer heating element and exposed to 70% heat intensity for 50 s. The heating element was removed, and the mold was immediately brought up until the mold platform was flush with the substrate. A vacuum pressure of −0.9 bar was then applied to seal the substrate to the mold, and it was allowed to cool to room temperature before being removed from the mold. A schematic of the entire process (BNNT deposition, screen printing of the ink, and thermoforming) is shown in Figure 1. Temperature analysis of the substrate undergoing thermoforming was conducted using a Bokar XTC-Profiler fitted with six thermocouples and the corresponding XTC-Profiler software to correlate the set power intensity and corresponding maximum temperature that the substrate reaches under these conditions. The change in temperature over 50 s under 70% power is presented in Figure S1.

**Figure 1:**
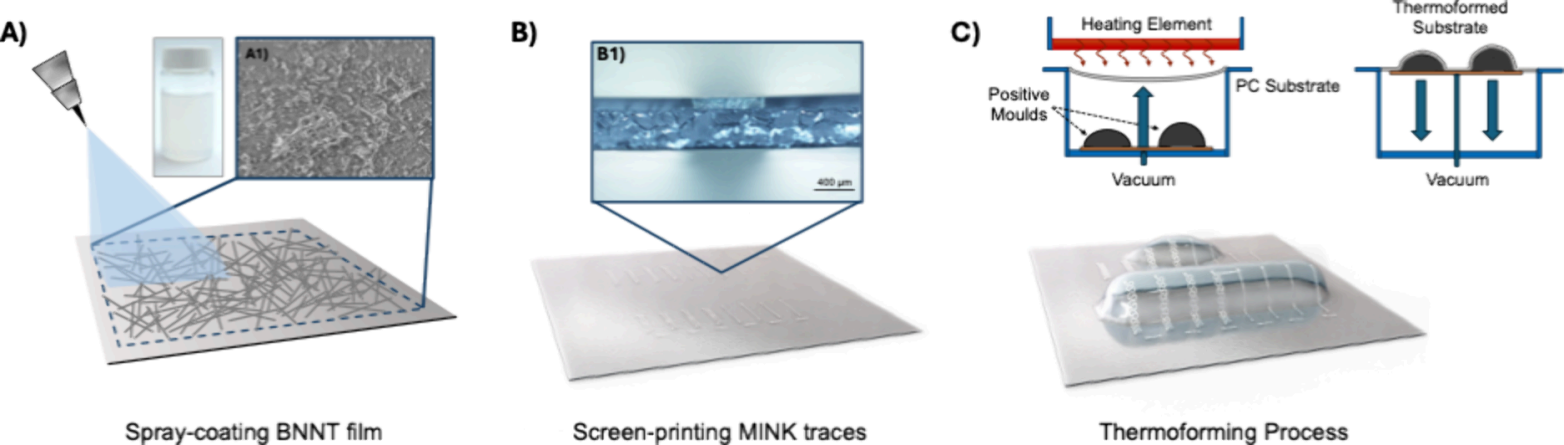
Schematic of (A) spray-coating of the BNNT suspension onto bare PC substrates with (A1) an inset SEM image of the BNNT film, (B) 2D-screen-printing technique used to deposit the MINK onto the PC substrate with (B1) an inset of a cross-sectional microscope image of a silver trace printed on a BNNT-coated PC substrate, and (C) thermoforming of the traces into 3D spherocylinder forms.

### Characterization

Resistance measurements were gathered using a Fluke 177 True RMS multimeter. Measurements were taken from the printed electrical pad to ensure proper contact between the probes and trace. The extent of elongation was manually measured across 25 data points using a ruler with a resolution of 0.5 mm to assess the change in length upon thermoforming. Current-carrying capacity (CCC) testing was completed using a Global Specialties 1325 voltmeter attached to the multimeter and alligator clips attached to the printed electrical pads to apply current. Starting at 1.0 V, a step increase of 1.0 V was applied to the traces every 60 s until the traces were no longer able to pass current and became nonconductive. Once stable, the measured current at each step increase was also recorded. A thermal camera (Jenoptik Optical Systems) was used to capture the heat map images, taking photographs every 1 s for the duration of the CCC measurements and then until the traces returned to room temperature. A Nikon SMZ800 DS camera stereomicroscope fitted with a MKII fiber-optic light was used to obtain surface images of the traces post-thermoforming, and an SU3500 scanning electron microscope gathered images of the nanotube interlayer and high-magnification images of the traces post-thermoforming.

## Results and Discussion

Thermoforming allows for the fabrication of 3D electrical interconnects from 2D thermoplastic substrates in a simple, accessible, low-cost, and scalable manner combining high-throughput screen printing and thermoforming processes. The challenge in realizing this technology is translating the 2D substrate to a 3D shape, where the printed components and substrate must both stretch to accommodate the change in geometry.^24^ It is therefore important to understand how the desired shape and angles of the molds impact the electrical properties of the elongated printed features on the 3D object, particularly their effect on the CCCs of the stretched features. The highest point of elongation, located where the substrate physically transitions from 2D to 3D, is a typical failure point in thermoformed traces by using commercially available inks designed for these applications. The ability for traces made with MINKs to maintain end-to-end conductivity upon thermoforming (even at elongations as high as 50%) highlights it as an ideal material for thermoforming applications. Combining MINKs with the BNNT interlayers should increase the mechanical and thermal stability of the conductive traces and subsequently improve their electrical performance post-thermoforming.

As thermoforming angles and the height of the 3D feature increase, printed traces tend to become less conductive due to subsequent thinning or cracking of the trace at the points of extreme elongation as the substrate is stretched more significantly. Therefore, it is important to correlate the thermoforming angle with substrate elongation upon thermoforming.^25^ To better understand how the shape and height of the mold stretch the PC substrate, bare films were subjected to thermoforming angles of 17.5, 35.0, and 70.0° using spherocylindrical shapes. As shown in Figure 2A, a 5.0 cm × 20.0 cm rectangular grid was hand-drawn with 0.2 cm × 0.2 cm squares on a bare PC substrate in the area where the traces will be printed. The marked substrate was then thermoformed, and the change in the square width was measured to collect percent elongation data. Mold angles of 17.5 and 35.0° display changes in percent elongation of up to 13.3% and 23.3%, whereas a mold angle of 70.0° begins to show significant change in percent elongation, reaching up to 36.7%. This is further visualized in Figure 2B,C which show a 3D rendering of the change in percent elongation at 70.0, 35.0, and 17.5°, respectively, as well as a quantified plot detailing the change in percent elongation in Figure 2D. This range provides insight into the level of deformation that the traces undergo, particularly at the 2D-to-3D transition point, where the percent elongation is the highest and where conductivity is likely to be most impacted. In addition to varying the thermoforming angle, two different trace designs, linear traces with a nominal line width of 10 mil (254 μm) and serpentine traces with a nominal line width of 20 mil (508 μm), were subjected to each thermoforming angle deformation. Serpentine lines have been demonstrated to better accommodate natural deformation under stretching than linear traces because they can twist and buckle under strain. These different trace geometries will provide a good comparison of how different trace geometries impact thermoforming.^26^

**Figure 2:**
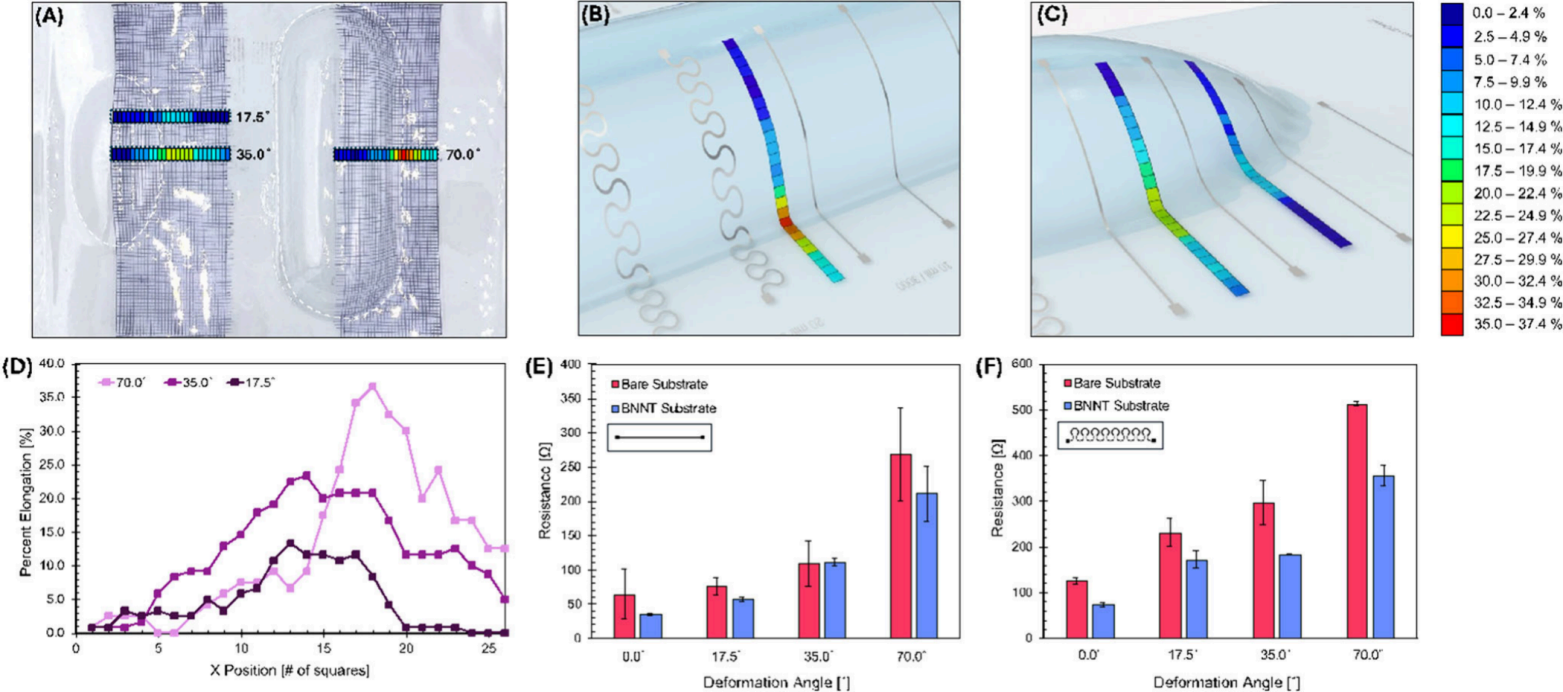
(A) Photograph highlighting the points of elongation measured. Schematic visualizations of (B) 70° and (C) 35 and 17.5° samples. (D) Percent elongation calculated using a running average, (E) resistance measurements of straight traces, and (F) serpentine traces subjected to each elongation angle with standard deviation error bars.

### Conductivity Analysis

During the thermoforming process, the substrate is stretched, and the point of highest elongation is created at the junction, transitioning from the flat substrate to the curved substrate. It is also noteworthy that the extent of elongation increases as the angle and height of the mold increases. The electrical resistance is measured for both linear and serpentine traces after being subjected to thermoforming over molds with 17.5, 35.0, and 70.0° mold angles, and the data are presented in Figure 2E,F, respectively. As shown with the linear traces, resistance values are similar both with and without the BNNTs present at lower mold angles; however, with a 70.0° deformation, the traces on the BNNT interlayer have lower resistances than those without the BNNT interlayer. Corresponding standard deviations on the resistance measurements also show a smaller discrepancy when BNNT interlayers are present. In a similar result, the serpentine traces exhibit overall lower resistance values with BNNTs present; however, the deviation is more similar than with the linear traces. Interestingly, the benefit of using BNNTs is statistically significant with mold angles of 35.0 and 70.0° when using serpentine traces, whereas it is only the case for 70.0° when using linear traces. In previous work, the inclusion of the BNNT interlayer has been demonstrated to alleviate mechanical stress imparted on 2D-screen-printed silver MINK traces, enabling them to better withstand repeated flexing/bending with little change in resistance.^19,23^ We suggest that the lower resistance values measured for the thermoformed traces on the BNNT layer in comparison to those on the bare substrate may also be due to this improved mechanical stability. We speculate that the BNNT interlayer provides a physical separation between the traces and the substrate that buffers the strain exerted on the traces following elongation in a manner similar to that demonstrated for the 2D traces, allowing the trace to stretch with less impact on the trace resistance.

To better understand what morphological changes occur in the traces during the thermoforming process, the correlation between the electrical resistance and the thickness of the substrate is explored in Figure 3. The resistance of the trace was measured in 2.5 mm intervals totaling 15 sections across the length of each trace, and the change in the resistance between each section was calculated and recorded. A digital caliper with an accuracy of 0.01 mm was used to measure the change in the substrate thickness, where 100% was the substrate’s nominal width of 0.5 mm and subsequent percentage decreases represent the remaining substrate thickness post-thermoforming. Parts A and B of Figure 3 provide a comparison for linear traces on bare and BNNT-coated traces, respectively, thermoformed over the mold with 70° angles to best highlight the effects of thermoforming on the traces. A clear correlation between the change in resistance and substrate thickness exists and illustrates that the traces are most affected in the areas that undergo the greatest elongation. This is particularly evident in the areas where the substrate thickness falls below 85% of its original thickness and the resistance reaches a maximum. While the change in electrical resistance is similar between the linear traces, there is an improvement in the delta change in resistance for serpentine traces printed on BNNT-coated PC against a bare PC substrate, as shown in Figure 3C,D.

**Figure 3:**
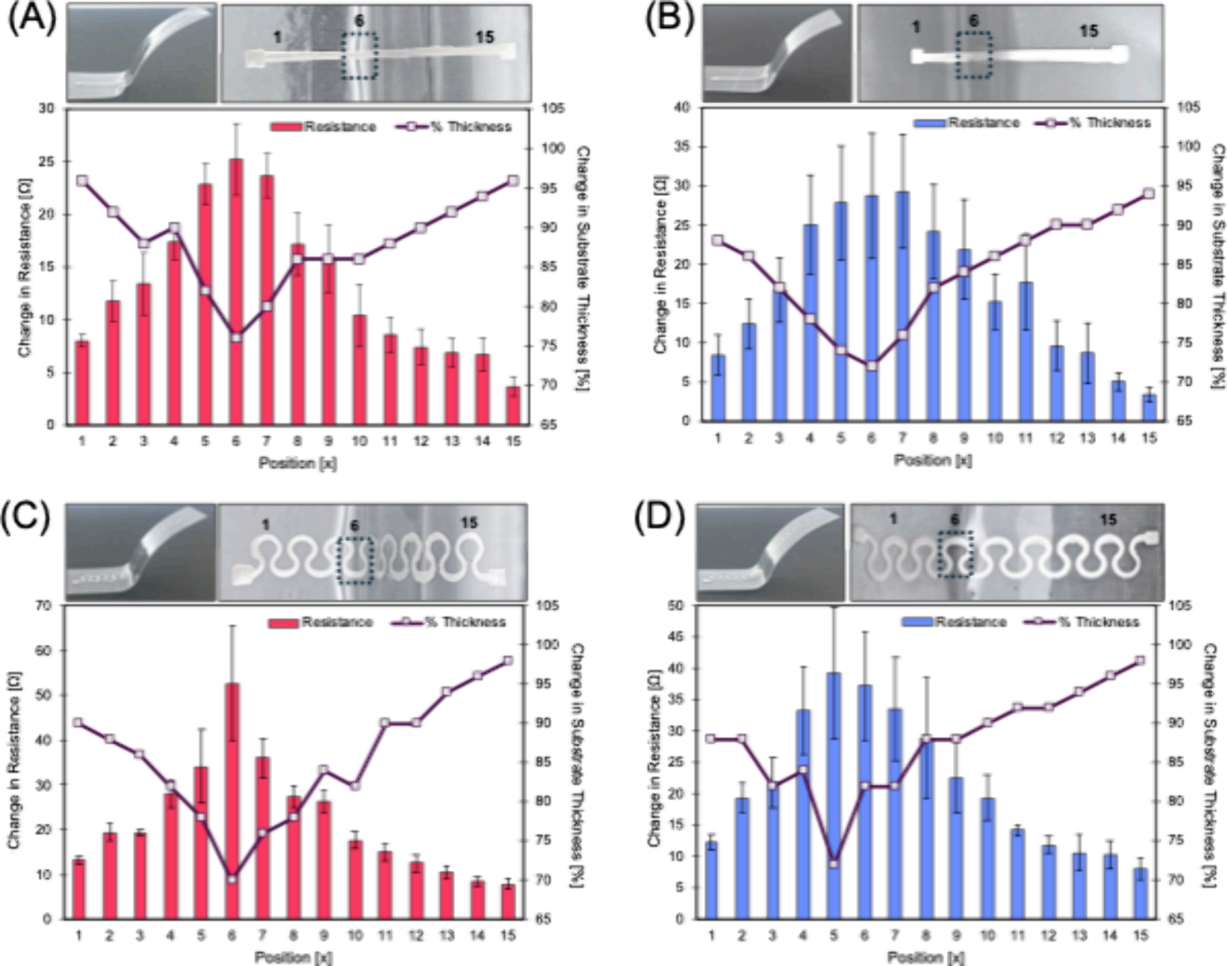
Comparison of the change in resistance across the trace length against thinning of the substrate upon thermoforming for linear traces on (A) bare and (B) BNNT-coated substrates and serpentine traces on (C) bare and (D) BNNT-coated substrates. The sample population for the standard deviation calculation is *n* = 5 for each figure.

Scanning electron microscopy (SEM) images in Figures S2 and S3 highlight the BNNT film with no trace as well as the elongation point of the linear trace on the BNNT network after thermoforming compared with the bare substrate, where the BNNTs can be observed through the silver ink pores that are present in the traces. These images demonstrate how the BNNTs are well distributed below the traces, which can help to provide bridges when the ink is thinned at the inflection point. Furthermore, Figures S4 and S5 show microscope images of the thermoformed linear and serpentine traces measured in Figure 3, shown on either side of the elongation point, particularly the flat section and curved edges. These images confirm no definitive damage across the areas of the trace, indicating that the deformation is localized to the junction point, as shown in the SEM image.

### CCC Testing and Thermal Analysis

There are several examples in the literature where functional 3D devices have been produced through thermoforming.^13,27^ These applications typically require quite low currents such as powering light-emitting diodes (LEDs), so the localized increases in resistance caused by thermoforming do not negatively impact the device performance. However, it is of interest to understand how thermoforming affects the electrical properties of the traces as increasingly higher currents are applied to them. This is relevant for developing more advanced IME applications that require demanding electrical performance, such as heaters and printed power lines to replace wires for driving devices requiring more power/higher current than simple LEDs. To understand how thermoforming impacts the electrical performance of the traces, CCC measurements were conducted on the 3D traces on both bare PC and traces deposited on the BNNT-coated PC. CCC measurements characterize how much current a conductor can withstand before it becomes nonconductive. In the context of printed electronics, the loss of conductivity is typically caused by the underlying plastic substrate warping or, more drastically, melting, causing a deformation that severs the conductor.^28^ CCC measurements are typically recorded for traces printed on plastic substrates, and while printing MINKs in 2D produces traces with uniform trace thickness over the entire length of the traces, the thinning of the trace observed upon thermoforming introduces nonuniformity that can affect the maximum current that can be carried by 3D traces.

During CCC testing, voltage is applied to a trace, leading to the current increasing and subsequently the temperature of the trace increasing due to Joule heating, which is monitored by using an IR thermal camera. The resulting temperature uniformity of the traces can be measured and recorded to determine whether the regions where the trace is thinner will create localized temperature increases (i.e., hot spots). It is likely that the formation of localized hot spots will be a point of failure (POF) and an indication that the CCC has been reached. Figure 4 presents the CCC measurements on a straight and flat 2D trace acting as a control and compares them to a straight and serpentine trace elongated over a spherocylinder mold with a 70.0° angle, as well as the corresponding maximum temperature at each applied voltage. It should be noted that traces with similar resistances were chosen to isolate the effects of the BNNT interlayer. Traces were subjected first to 1.0 V, which was then increased at increments of 1.0 V held for 60 s each, until the measured current fell to 0 mA, indicating that the traces were no longer conductive. In this context, the CCC is defined as the ability for the trace to maintain its current for the entirety of its 60 s interval. Furthermore, the POF is defined as the highest recorded current 1 s before the trace fails. The voltage value presented for the POF is higher than that of the reported CCC, which has a voltage at which the trace failed prior to completion of the 60 s interval. Figure 4 shows thermal images corresponding to the images taken during the CCC tests, providing insight into the thermal profile of the printed traces on bare and BNNT-coated substrates as the voltage/current is increased, as well as the measured maximum temperature of the traces before the POF. As expected, when 2D traces of the MINK are characterized both on bare PC and over the BNNT interlayer in Figure 4A, the CCC and resulting temperature of the traces are uniform and can reach temperatures close to the *T*_g_ value of the PC substrate (*T*_g_ ≈ 140 °C), at which time the traces burn out because the underlying substrate deforms and the traces become nonfunctional. These traces do not typically have specific locations where they fail. Here the entire trace tends to melt into the substrate before cracks form and it fractures. Consistent with our previous reports, the maximum temperature that the traces can reach before melting is higher for the traces printed on the BNNT interlayer than on the bare PC substrate. Additionally, the CCC of the control traces printed on PC is almost identical to the BNNT samples until 5 V, where the CCC and maximum temperature for the trace printed on BNNT-coated substrates begin to increase above those printed on bare substrates. This phenomenon can be attributed to the BNNTs assisting in ohmic sintering of the traces, where a decrease in the resistance of the traces upon increasing applied voltage was measured, which ultimately led to a higher temperature output.^19^ We reported this previously for a similar MINK printed on a BNNT interlayer on PET substrates.

**Figure 4:**
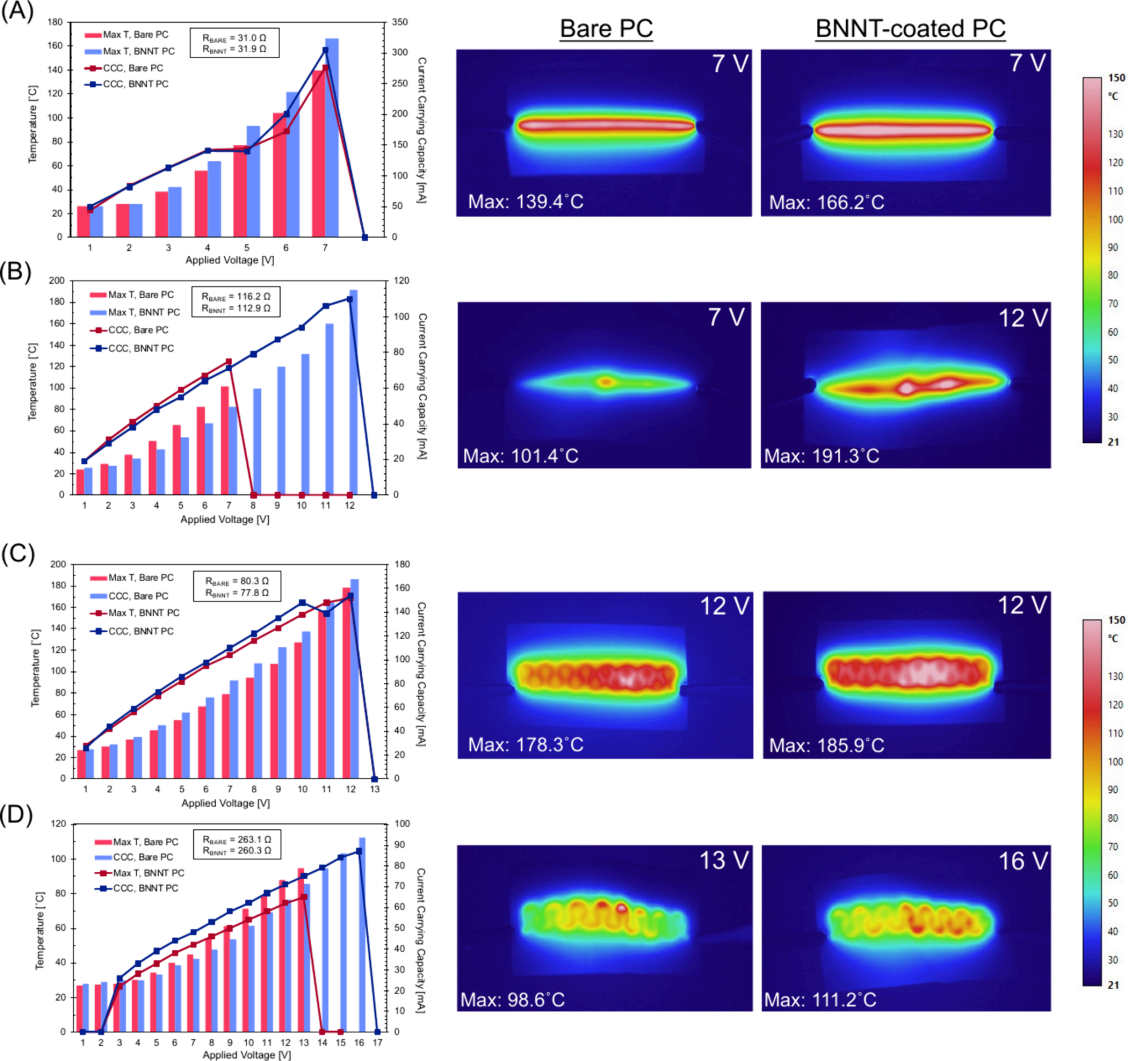
CCC and corresponding temperature response at increasing applied voltage for straight line traces on (A) a flat substrate and (B) thermoformed with a spherocylinder-shaped mold with a 70.0° angle, and serpentine traces on (C) a flat substrate and (D) thermoformed with a spherocylinder-shaped mold with a 70.0° angle printed on bare PC and BNNT-coated PC substrates. The applied voltage was maintained for 60 s before increasing to the next voltage, at a step size of 1.0 V.

Figure 4B shows the CCC measurements for the straight trace printed on bare PC and those printed on PC with a BNNT interlayer and thermoformed using a mold with 70° angles. For both sets of traces, as the voltage/current increases, there is a significant difference in the thermal properties of the thermoformed 3D traces in comparison to the 2D traces. As shown in Figure 3, thermoforming produces localized areas of higher resistance at the site of elongation for the 3D traces. It has been previously reported that the electron flow (i.e., current) is less efficient through areas of higher resistance, and this area of high resistance/restricted electron flow correlates to the location of this localized temperature increase. It is noteworthy that the 3D traces on the BNNT interlayer show improvements in both the CCC and the ability for the substrate to withstand temperature increases that occur when high currents are applied. In a comparison of the traces of similarly measured resistance, the trace printed on BNNT-coated substrates outperforms the trace without, leading to operation up to 13 V, whereas the traces on bare substrates reach their CCC at 9 V. Furthermore, the thermal conductivity of the BNNTs assists in reducing the temperature accumulation at the most significant elongation point, as is evident by the lower maximum temperature at each applied voltage until failure. This can be attributed to the BNNTs’ acting as buffering layers to dissipate generated heat across the nanotube intersections and away from the trace, so the PC substrate no longer softens and deforms the silver trace, instead allowing the trace to operate at higher currents/temperatures before failing. A similar comparison is done on 2D serpentine traces (Figure 4C) that have also been thermoformed with a spherocylinder-shaped mold with a 70.0° angle in Figure 4D. The results of the CCC measurements for 2D serpentine traces both on bare PC and over the BNNT interlayer in Figure 4C show that the temperature of the traces are uniform and can reach temperatures close to the *T*_g_ value of the PC substrate (*T*_g_ ≈ 140 °C) at which time the traces burn out because the underlying substrate deforms and the traces become nonfunctional. While not as pronounced as that for the flat traces in Figure 4B, a similar trend is observed with the thermoformed serpentine traces, where those printed on BNNT-coated substrates exhibit lower maximum temperatures at the inflection point and ultimately can withstand more current, failing at 15 V, whereas the bare substrate counterparts fail at 13 V (Figure 4D). The CCC values and operating temperature/voltage are improved using BNNT interlayers in both 2D- and 3D-printed silver traces.

Further insight into the POF, found in Figure 5, is shown through the corresponding trace tested in Figure 4B. Because the highest point of elongation causes a localized increase in the electrical resistance, the areas of high resistance will cause a higher temperature output. A significant temperature increase is present in the region of highest elongation in the trace, and as previously mentioned, exceeding the *T*_g_ value of the underlying substrate will result in a deformation and loss of current because the traces fracture. Figure 5A, a photograph of the linear trace thermoformed on bare PC, shows clear deformation/melting of the substrate, leading to cracks that run across the width of the trace. Notably, the damage is localized to this area only, and no adverse changes to the trace morphology on either side of the hot spot are visible. This once again demonstrates that it is only the point of elongation (and localized high resistance) that causes the temperature increase and trace failure during the CCC experiment for these 3D traces. Similar images in Figure 5B are produced for the linear trace thermoformed on the BNNT-coated substrate, showing significant warpage/deformation locations overlapping the thermal camera images and mapping the highest temperatures with the areas of high elongation, as expected. While both sets of traces fail, the traces thermoformed with the BNNT interlayer ultimately fail at higher currents and corresponding higher temperatures because the BNNT network is able to redistribute the generated heat away from the hot spot throughout the nanotubes before affecting the substrate. Therefore, the trace can withstand higher temperatures before failure, even when the traces exhibit near-identical resistance values.

**Figure 5:**
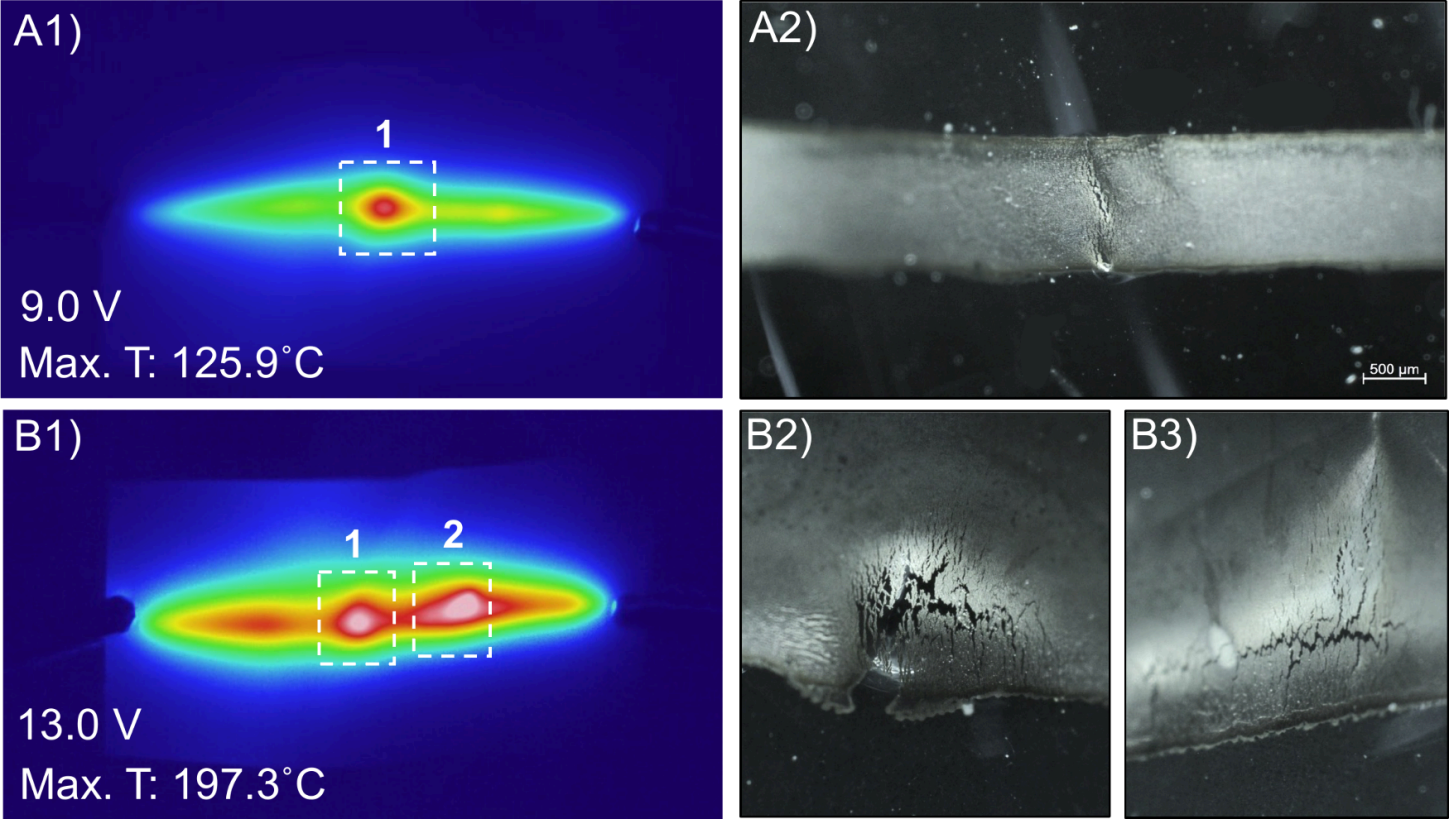
POF images of a linear trace thermoformed with a spherocylinder-shaped mold with a 70.0° angle on a bare PC substrate with the (A1) thermal image and (A2) corresponding microscope image of the failure point. POF images on the BNNT-coated substrate of the (B1) thermal image and (B2 and B3) corresponding failure point microscope images.

## Conclusions

We successfully demonstrated the utility of BNNT interlayers for improving the electrical performance of thermoformed 3D traces. We show that regions of high elongation across thermoformed traces cause adverse effects to the CCC of the trace due to substrate/trace thinning, resulting in localized areas of high electrical resistance and leading to drastic temperature increases when high currents are applied to the traces. BNNT interlayers assisted in reducing the initial electrical resistance of both linear and serpentine thermoformed 3D traces at nearly all explored thermoforming angles, likely acting as buffer layers as the traces are stretched. Additionally, thermal dissipation properties of the BNNT network delayed deformation of the underlying substrate caused by heat generated in the trace when subjected to high operational voltages/currents, leading to an increase in the CCC of the thermoformed traces when the BNNT interlayer was present. Overall, this study demonstrates the use of BNNT interlayers to improve the electrical performance of 3D silver MINK traces employed in IME applications using thermoforming, where significant improvements in the thermal management properties of the traces allow the substrate to exceed its glass transition temperature without failure.

## Supplementary Material



## Notes

Equipment calibration data and qualitative imaging of the BNNT film and silver traces (PDF)
