# Supplementary material for: Improving Printed and Thermoformed Conductors on Polycarbonate with a Thin-Film BNNT Interlayer for Next-Generation In-Mold Electronics
Source: ACS Appl Mater Interfaces. 2025 Sep 9;17(38):54157–65. doi: 10.1021/acsami.5c07261 (PMC12464897; doi:10.1021/acsami.5c07261)
Supplement: Supplementary file 1 [file am5c07261_si_001.pdf]

Improving printed and thermoformed conductors on polycarbonate with a thin film BNNT interlayer for next-generation in-mold electronics

Kaitlin Wagner,[1,2] Arnold J. Kell,[2*] Xiangyang Liu,[2] Liliana Gaburici,[2] Joseph Manion,[1] Chantal Paquet,[2] and Benoît H. Lessard,[1,3*]
[1] Chemical and Biological Engineering, University of Ottawa, 161 Louis Pasteur, Ottawa, ON K1N 6N5, Canada
[2] Quantum & Nanotechnologies Research Centre, National Research Centre Canada, 100 Sussex Drive, Ottawa, ON, K1A 0R6, Canada
[3] School of Electrical Engineering and Computer Science, University of Ottawa, 800 King Edward Ave, Ottawa, Ontario K1N 6N5, Canada

Corresponding Authors:
Email: Arnold J. Kell, arnold.kell@nrc-cnrc.gc.ca
Email: Benoît H. Lessard, benoit.lessard@uottawa.ca

Electronic Supporting Information

Table S1. Spectral data for one pass under both the iron and gallium lamps set to medium intensity at 30 FPM was measured using an EIT UV Power Puck II radiometer

Light Wavelength [-]	[mJ/cm^2]	[mW/cm^2]
UVA	767.170	1327.328
UVB	529.607	659.086
UVC	137.968	169.414
UVV	1833.184	2969.880

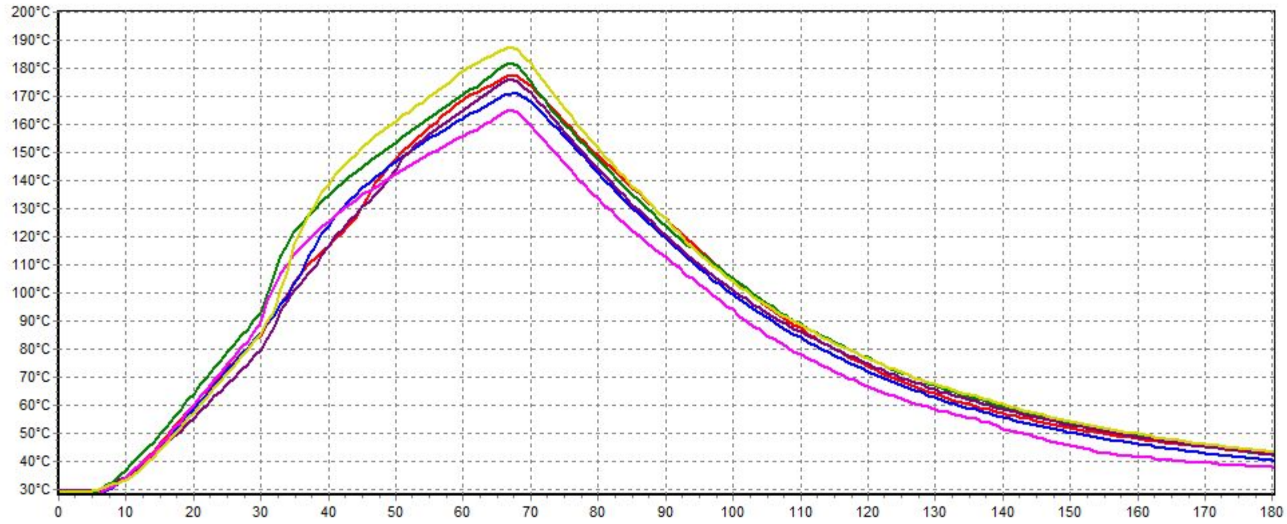

Figure S1. Change in temperature at six areas of a 450DT thermoformer, each set to 70% power and exposed for 60s. Measured and plotted using Bokar XTC-Profiler and compatible XTC-Profiler software.

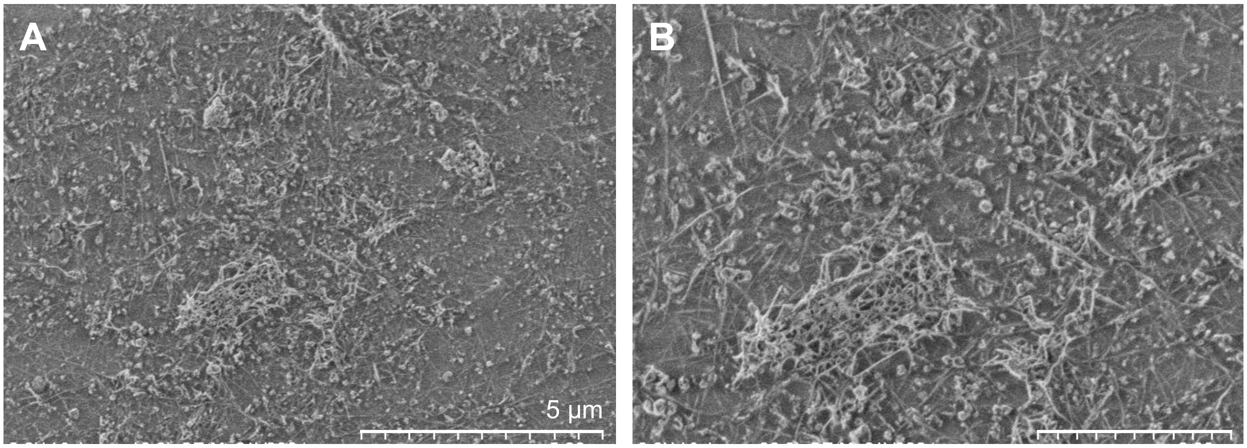

Figure S2. SEM images of the 6-layer thick BNNT film on a polycarbonate substrate shown at (A) 10.0k and (B) 30.0k magnifications.

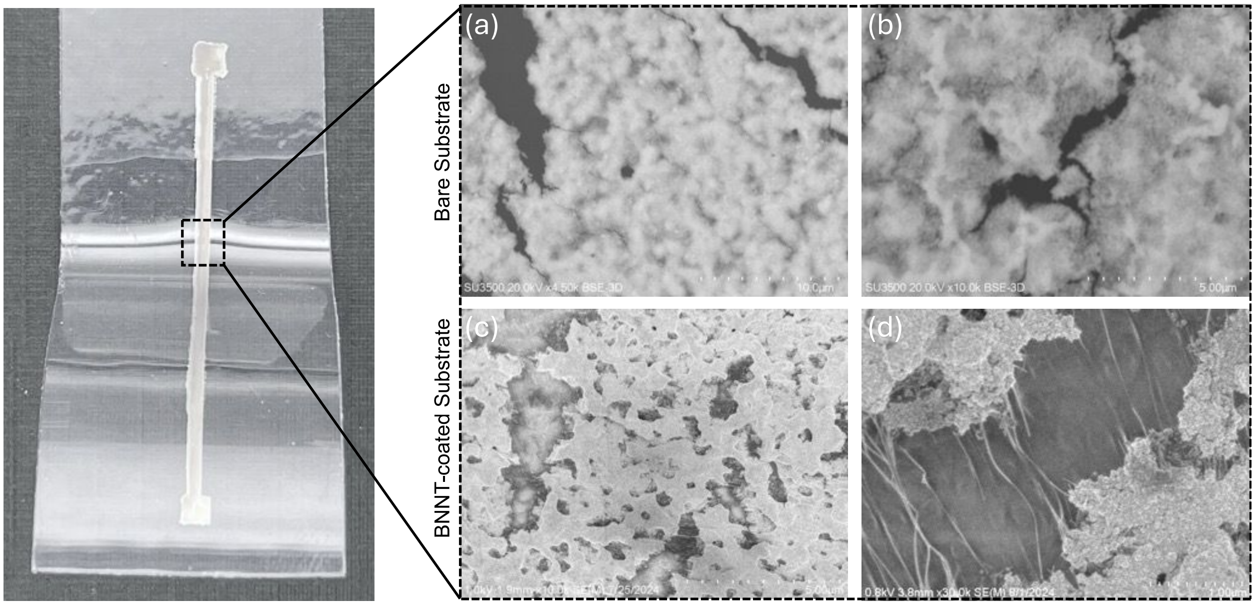

Figure S3. SEM images of linear 70˚ thermoformed trace taken at the highest point of elongation, on a bare substrate at (a) 4.50k, and (b) 10.0k magnification, and the trace printed on 6-layer thick spray-coated boron nitride nanotubes on PC substrates, BNNT-coated substrates, highlighting the nanotube network underneath at (c) 10.0k, and (d) 30.0k magnification

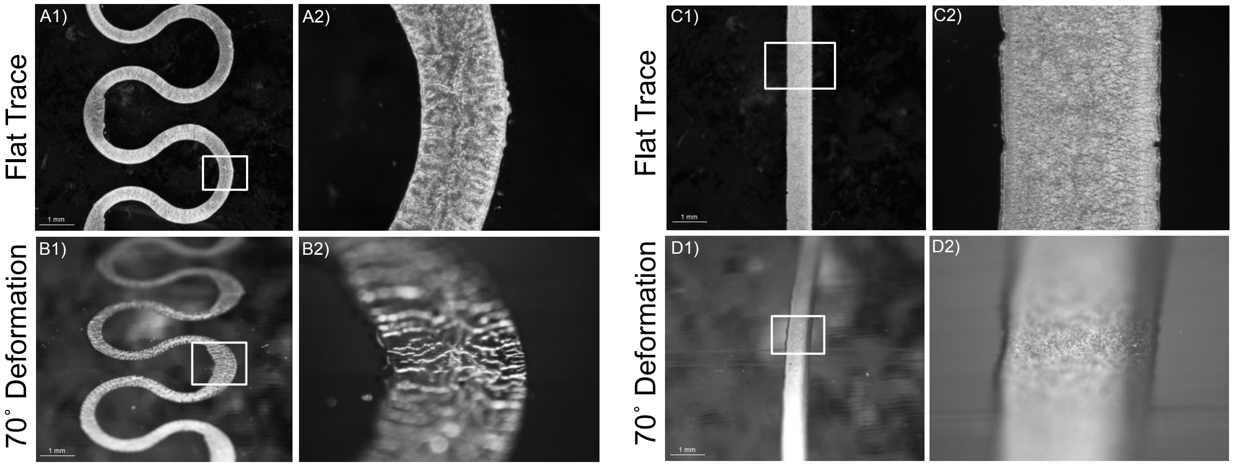

Figure S4. Microscope images of Ag MINK printed on bare PC substrates, comparing trace quality after thermoforming on a flat surface or subjected to 40% elongation, or 70˚ deformation. Serpentine traces are shown A) on the flat surface of the substrate and B) along the curved edge of the 3D feature. Straight traces are shown C) on the flat surface of the substrate and D) along the curved edge of the 3D feature. Images denoted with a ‘1’ represent a magnification of 1.0X, and ‘2’ represents a magnification of 6.3X for all presented images.

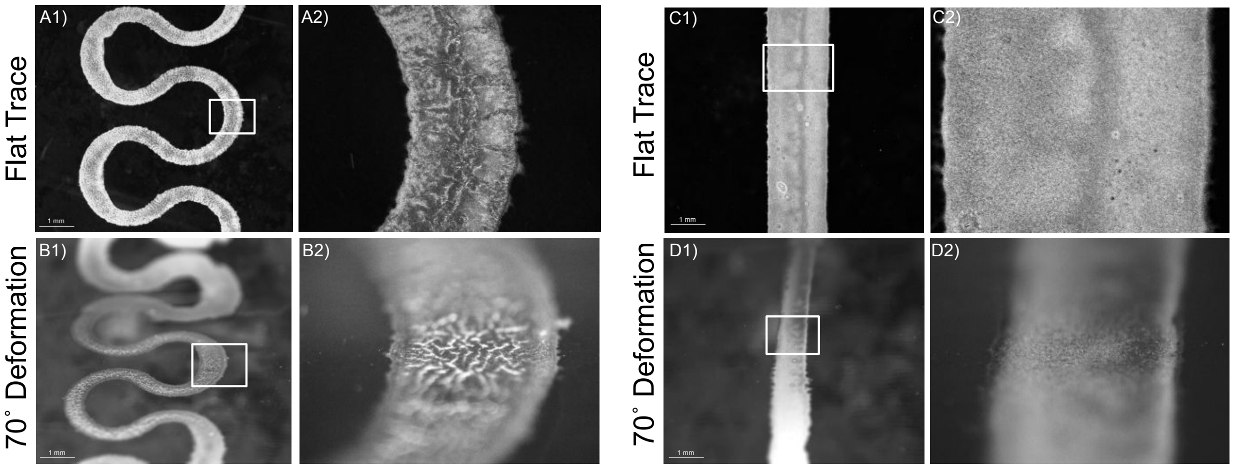

Figure S5. Microscope images of Ag MINK printed on BNNT-coated PC substrates, comparing trace quality after thermoforming on a flat surface or subjected to 40% elongation, or 70˚ deformation. Serpentine traces are shown A) on the flat surface of the substrate and B) along the curved edge of the 3D feature. Straight traces are shown C) on the flat surface of the substrate and D) along the curved edge of the 3D feature. Images denoted with a '1' represent a magnification of 1.0X, and '2' represents a magnification of 6.3X for all presented images.
